# Endothelial to mesenchymal Notch signaling regulates skeletal repair

**DOI:** 10.1172/jci.insight.181073

**Published:** 2024-05-23

**Authors:** Sanja Novak, Hitoshi Tanigawa, Vijender Singh, Sierra H. Root, Tannin A. Schmidt, Kurt D. Hankenson, Ivo Kalajzic

**Affiliations:** 1 Center for Regenerative Medicine and Skeletal Development, School of Dental Medicine, UConn Health, Farmington, Connecticut, USA.; 2 Institute for Systems Genomics, Computational Biology Core, UConn, Storrs, Connecticut, USA.; 3 Biomedical Engineering Department, School of Dental Medicine, UConn Health, Farmington, Connecticut, USA.; 4 Department of Orthopaedic Surgery, University of Michigan Medical School, Ann Arbor, Michigan, USA.

**Keywords:** Bone biology, Growth factors, Orthopedics

## Abstract

We present a transcriptomic analysis that provides a better understanding of regulatory mechanisms within the healthy and injured periosteum. The focus of this work is on characterizing early events controlling bone healing during formation of periosteal callus on day 3 after fracture. Building on our previous findings showing that induced Notch1 signaling in osteoprogenitors leads to better healing, we compared samples in which the Notch 1 intracellular domain is overexpressed by periosteal stem/progenitor cells, with control intact and fractured periosteum. Molecular mechanisms and changes in skeletal stem/progenitor cells (SSPCs) and other cell populations within the callus, including hematopoietic lineages, were determined. Notably, Notch ligands were differentially expressed in endothelial and mesenchymal populations, with *Dll4* restricted to endothelial cells, whereas *Jag1* was expressed by mesenchymal populations. Targeted deletion of *Dll4* in endothelial cells using Cdh5CreER resulted in negative effects on early fracture healing, while deletion in SSPCs using α-smooth muscle actin–CreER did not impact bone healing. Translating these observations into a clinically relevant model of bone healing revealed the beneficial effects of delivering Notch ligands alongside the osteogenic inducer, BMP2. These findings provide insights into the regulatory mechanisms within the healthy and injured periosteum, paving the way for novel translational approaches to bone healing.

## Abstract 2

Characterization of early events in bone healing indicates that endothelial to mesenchymal Notch signaling regulates skeletal repair through Dll4 signaling.

## Graphical abstract

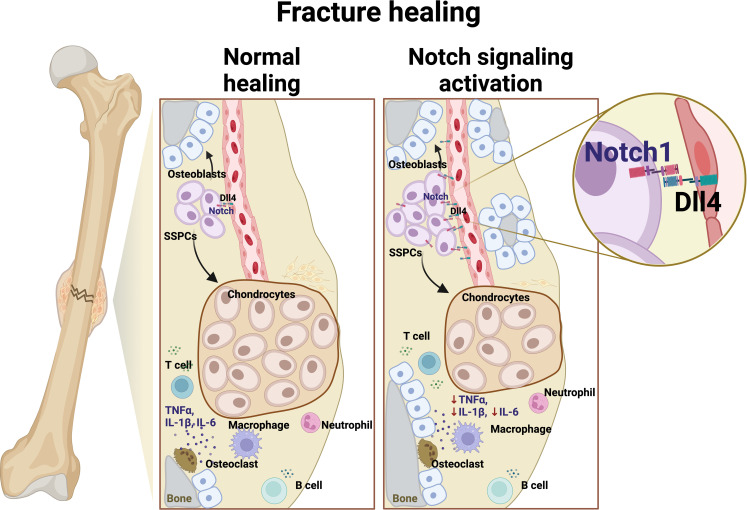

## Introduction

Regeneration of the skeleton depends on various factors, including skeletal stem/progenitor cells (SSPCs) and their interactions with other cell populations in the periosteal and bone marrow niche. Fractures cause damage to bone and surrounding tissues, causing bleeding, hematoma formation, and an influx of hematopoietic cells to the fracture site. These events lead to the expansion of SSPCs and endothelial cells (ECs). Previous studies from our lab and other groups have shown that periosteum is a major contributor to fracture healing (1–3). The strong evidence supporting the periosteum as major contributor to fracture healing was recently reported by genetic lineage tracing studies published by Liu et al. (4).

A critical event controlling tissue repair is whether SSPCs undergo proliferation or differentiation. During this early phase of fracture healing, autocrine and paracrine signals direct SSPC fate toward commitment to chondrogenic and osteogenic lineages. However, the molecular pathways and cell-to-cell signaling mechanisms controlling SSPC activation in the cellularly heterogeneous callus remain poorly understood.

Notch signaling is a conserved pathway with roles in development, homeostasis, and tissue regeneration (5). This pathway plays a major role in maintaining progenitor pools and controlling differentiation into mature lineages across various cell types (6). The actions of Notch signaling are divergent and temporally controlled, exhibiting different effects depending on the specific tissue and stage of cell lineage maturation (5, 7). Notch signaling depends on the binding of Notch ligands (Jagged 1 and 2 [JAG1 and -2] and Delta-like ligand 1, 3, and 4 [DLL1, -3, and -4]) to Notch receptors (Notch1–4) (5, 6). Upon receptor-ligand binding, a conformational change in the receptor facilitates γ-secretase cleavage of the Notch receptor intracellular domain (NICD). The NICD then binds with recombination signal binding protein for immunoglobulin κ J region (Rbpjκ) and Mastermind-like (MAML) proteins, inducing gene transcription. This signaling sequence is commonly referred to as canonical Notch signaling. However, Notch also signals through noncanonical pathways that operate independently of Rbpjκ. In bone, the functions of Notch receptors are primarily nonredundant (5). Notch1 induces osteoprogenitor proliferation and inhibits mesenchymal stem cell (MSC) differentiation and osteoclastogenesis (8, 9), whereas Notch2 and Notch3 induce osteoclastogenesis (10). Deletions of Notch1 and Notch2 in Prx1-expressing MSCs lead to a high bone mass phenotype in adolescent animals, yet aged mice exhibit osteopenia due to depletion of the osteoprogenitor pool (11). Studies have shown that active Notch signaling impairs chondrocyte differentiation and endochondral ossification (12, 13). While bone development and the healing process share many biological aspects, the specific roles of Notch ligands and receptors have been relatively understudied (14, 15). Notch signaling activation has been shown to be beneficial in skin injury healing by increasing proliferation, while its inhibition impairs healing processes (16, 17). Transplanting MSCs overexpressing JAG1 leads to improved skin wound healing in a diabetic mouse model (18). JAG1 in myofibroblasts promoted Notch signaling in hepatic progenitor cells that led to their biliary specification to cholangiocytes. This process was inhibited by use of DAPT, a γ-secretase inhibitor (19). Notch signaling is active in epithelial cells of the inflamed mucosa of colitis, which when inhibited significantly exacerbates the clinical course of DSS-colitis, with significant increases in goblet cell number, severe loss of the epithelial layer, and infiltration of inflammatory cells into the small intestine (20).

The role of canonical Notch signaling in fracture healing has been evaluated by deleting Rbpjκ using Prx1Cre. In this model, fractures do not heal adequately, resulting in nonunion of the fractured bone even after 42 days post-fracture (dpf) (21). Our previous work showed that overexpressing Notch 1 intracellular domain (NICD1) in α-smooth muscle actin^+^ (αSMA^+^) osteoprogenitors improved fracture healing, through the expansion of osteoprogenitors (αSMA^+^ cells) at the fracture site and induction of differentiation into osteocalcin^+^ osteoblasts. Notch1 overexpression also led to an increase in callus bone mass, rendering fractured bones mechanically stronger (22). Furthermore, inhibition of Notch1 signaling through systemically delivered Notch1 antibody impaired bone healing, producing a phenotype similar to that observed with Rbpjκ deletion in Prx1 cells (21, 22).

Recognizing the clear evidence indicating that perturbations in Notch signaling impact bone healing, the objective of this study was to examine Notch ligands and identify the cell populations expressing them. Previous studies have demonstrated a beneficial effect on intramembranous bone healing using recombinant JAG1 on collagen scaffolds in the healing of cranial and femoral defects in mice and rats (23). Additionally, in a model of intramembranous formation involving bone marrow ablation, animals with *Jag1* deletion in osteoprogenitors showed a decrease in bone formation (23). Considering the limitations of prior studies on the effects of Notch ligands, we used single-cell RNA sequencing (scRNA-seq) to evaluate signaling in cell populations involved in normal fracture healing and in cases of Notch1 overexpression. We hypothesized that early events during the healing process are critical for SSPC lineage determination. Our study focused on characterizing single cells on day 3 of fracture healing. By determining the cell populations expressing Notch ligands, we were able to specifically target the deletion of *Dll4* in ECs and demonstrate the requirement of EC *Dll4* expression for bone healing. Insights gleaned from our single-cell genomic analysis, combined with a genetic deletion approach, prompted us to evaluate the therapeutic potential of delivering Notch ligands alone or as an enhancement to clinically used osteogenic inducers such as BMP2.

## Results

### Transcriptome analysis of early fracture healing.

**Figure 1:**
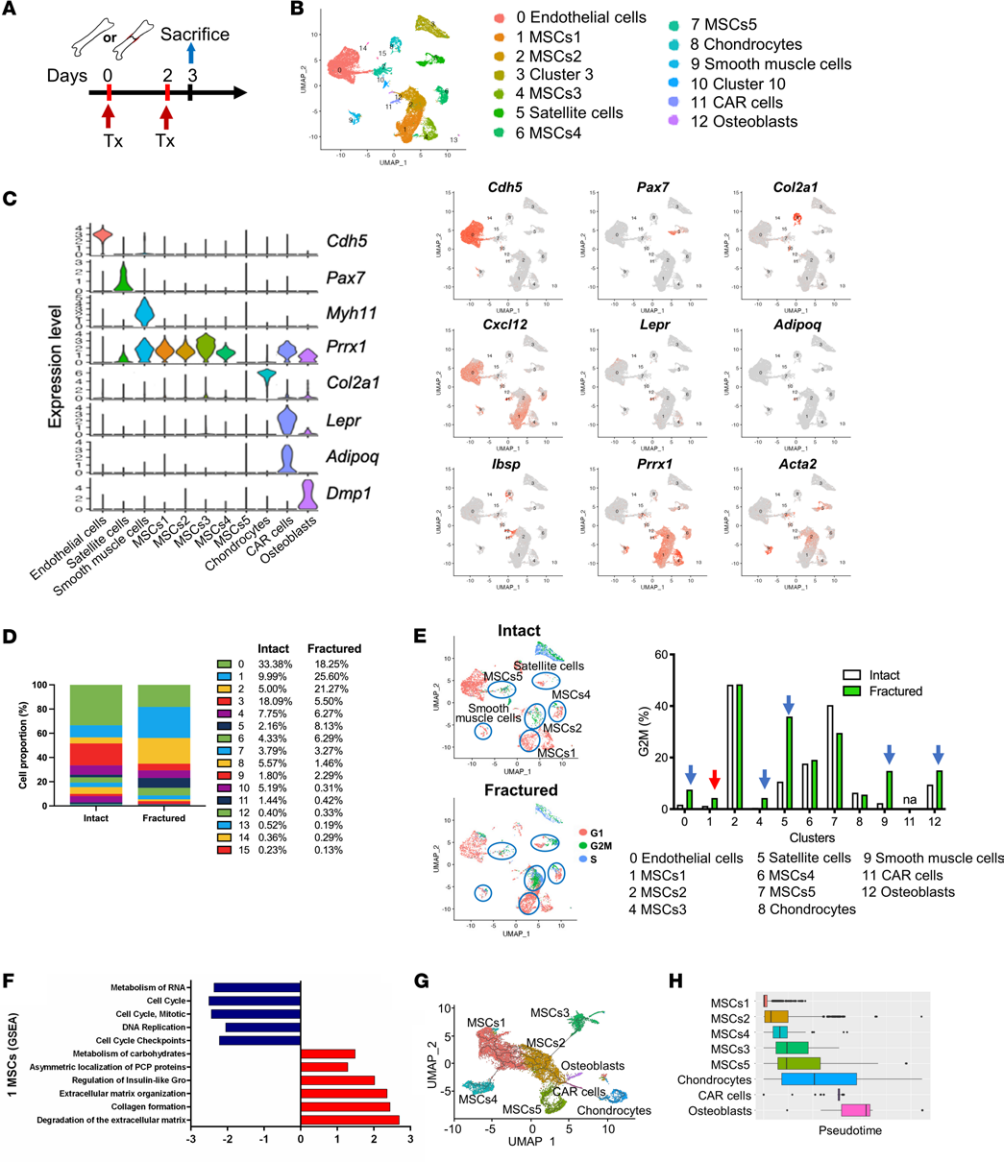
Transcriptional profiling of periosteal nonhematopoietic cells from intact and fractured bones. (**A**) Experimental design for mouse treatment aimed at collecting samples for scRNA-seq. Femur fractures were induced in SMACre^–^ER/NICD1 and SMACre^+^ER/NICD1 male mice. To induce overexpression of NICD1, animals were treated with tamoxifen (Tx) on the day of fracture and 2 dpf. Femur samples of intact or injured periosteum were collected, digested, and cells were sorted for CD45^–^ and CD45^+^. Subsequently, scRNA-seq was performed. (**B**) Clusters of periosteal CD45^–^ cell populations with (**C**) violin and feature plots presenting characteristic conserved gene expression for each cluster from integrated intact and fractured Cre^–^ and Cre^+^ samples are shown. (**D**) Proportion of cells within the control intact and fractured sample of each cluster. (**E**) Periosteal cells from Cre^–^ intact and fractured samples were analyzed for cell cycle phases and cell proportion in the G_2_M phase. (**F**) GSEA indicates that MSCs1 is a stem/progenitor cell population within the periosteum and (**G**) Monocle3 trajectory analysis (https://cole-trapnell-lab.github.io/monocle-release/) shows cell differentiation from the MSCs1 cluster to mature chondrocytes and osteoblasts from integrated intact and fractured Cre^–^ and Cre^+^ samples and (**H**) trajectory of the clusters in pseudotime.

Bones heal well in healthy individuals, but aging and confounding diseases can negatively impact this process (24–26). Despite our understanding of the critical role of osteogenic inducers in healing, little is known about the factors that regulate early events within the injured bone. Therefore, we focused our attention on day 3 following femoral fracture, as the expansion and commitment of SSPCs form the basis for the formation of mature lineages. To determine the potential molecular mechanisms regulating fracture healing, we performed scRNA-seq on periosteal cells isolated from intact and fractured bones (3 dpf) (Figure 1A). We sorted live, nonhematopoietic (CD45^–^) and hematopoietic (CD45^+^) cells (Supplemental Figure 1; supplemental material available online with this article; https://doi.org/10.1172/jci.insight.181073DS1) and performed scRNA-seq (10× Genomics).

Unsupervised clustering defined cell populations based on conserved gene expression in the clusters. Within the CD45^–^ periosteal populations, we identified 16 clusters, including ECs with characteristic expression of *Cdh5*, satellite cells expressing *Pax7*, and smooth muscle cells expressing *Myh11* (Figure 1, B and C). Several populations expressed *Prxx1* and were classified as MSCs, whereas more mature cells expressed typical genes; chondrocytes (*Col2a1*), reticular cells (*Adipoq* and *Lepr*), and osteoblasts (*Ibsp* and *Dmp1*) (Figure 1, B and C). We employed cell sorting to enrich MSC and EC populations within periosteal samples, although there was some contamination with hematopoietic cells (clusters 3 and 10). Clusters 13, 14, and 15 exhibited low numbers of cells (under 25 cells) and were therefore not analyzed (Figure 1D). Periosteal cells from cluster 1 (MSCs1), cluster 2 (MSCs2), and cluster 5 (satellite cells) were expanded by day 3 after fracture (Figure 1D). However, ECs, MSCs1, MSCs3, satellite cells, smooth muscle cells, and osteoblasts exhibited an increasing proportion of cells in the G_2_M phase (Figure 1E). The MSCs1 cluster showed characteristics of quiescent stem cells, with a gene expression profile showing inhibition of cell cycle genes and mitosis (*Cenpm*, *Rpa2/2*, *Rfc3/4*, *Ccnb2*, *Cenpa*, and *Spc24*) as well as DNA replication (*Gins2/4*, *Pold3*, and *Anapc15*). Based on the gene expression profile and proliferative capacity before and after the fracture and gene set enrichment analysis (GSEA), we concluded that the least differentiated cluster is MSCs1, which was set as a root population in trajectory analysis. Based on a trajectory analysis, the MSCs1 cell population rapidly proliferated after fracture and transitioned into a highly proliferating MSCs2 cluster (Figure 1, E–G). GSEA showed that this MSCs1 cluster was increased in gene sets involved in asymmetric localization of planar cell polarity proteins (*Scrib*, *Psmf1*, and *E2f3*), extracellular matrix, collagen formation (*Col2a1* and *Col11a1*), and degradation of the extracellular matrix, indicating a potential stem cell population (Figure 1F). These cells differentiate through MSC intermediate stages into chondrocytes or osteoblasts, as indicated by a trajectory analysis (Figure 1, G and H).

**Figure 2:**
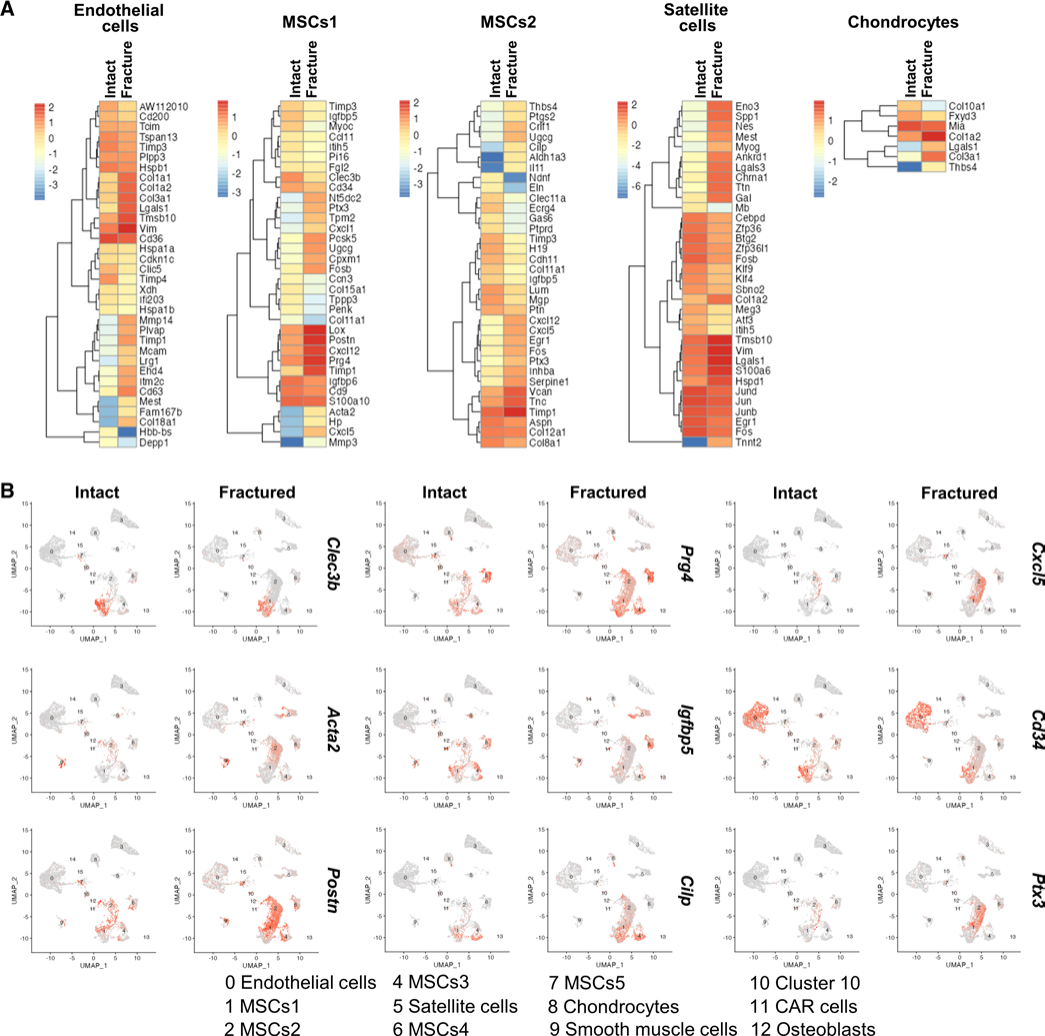
Periosteal cells from intact and fractured bones have distinct transcriptional profiles. (**A**) Heatmaps of EC, MSCs1, MSCs2, satellite cell, and chondrocyte clusters showing differentially expressed genes with significantly increased or decreased expression of Cre^–^ control fractured compared with intact samples. Color intensity represents the mean gene expression of all cells within the cluster. (**B**) Feature plots of the specific genes identified in **A** that were found to be significantly increased in periosteal cells upon fracture compared with intact samples.

Upon fracture, ECs showed higher expression of *Col18a1* (endostatin), *Plvap*, and genes encoding extracellular proteins like *Col3a1*, *Col1a2*, *Col1a1*, and *Col15a1* in comparison with the intact periosteum (Figure 2A). These genes are involved in the healing process (27). The MSCs1 cluster showed a significant increase in the *Cxcl5* chemokine, which recruits and activates leukocytes upon fracture. Genes that modulate the inflammatory response, such as *Prg4*, *Ptx3*, *Cxcl12*, *Cxcl1*, and *Il4ra*, were increased at the fracture site (Figure 2, A and B). With early tissue reorganization following injury, the expression of *Timp1* and *Mmp3* was increased. Many genes (e.g., *Acta2*, *Ptx3*, *Cxcl12*, *Il4ra*, and *Cxcl5*) were differentially expressed in all mesenchymal clusters of fractured samples compared with the intact periosteum. Only a few genes were differentially expressed in mature populations of chondrocytes (Figure 2A), whereas osteoblasts and CXCL12-abundant reticular (CAR) cells did not show any statistically significant differences in gene expression following the fracture. These mature populations were likely present at the time of a fracture, might have not gone through differentiation, and the bone injury did not significantly affect their transcriptional profile.

We performed CellChat (v2.1.2; https://github.com/jinworks/CellChat) analysis on our samples. More than 80% of signaling pathways in our samples (Cre^–^ and Cre^+^, both intact and fractured) had significant cell-cell interactions (activin, BMP, Notch, PDGF, IGF, MMP, and BSP) in the same signaling pathways (Supplemental Table 1). IL-6 signaling interactions were specific only to the control fractured Cre^–^ group. NEGR, SLIT, and WNT signaling pathways were specific pathways in the Cre^–^ intact sample compared with fractured Cre^–^ (Supplemental Figure 2A). IGF, collagen, BSP, VCAM, CXCL, PDGF, NOTCH, and periostin are some of the signaling pathways specific to the fractured compared with intact samples. In Notch interactions from ECs to other mesenchymal clusters of Cre^–^ intact and fractured samples, we found that EC communication probabilities were strongest among JAG2 and DLL4 ligands interacting with Notch2 and Notch3, while interactions with Notch 1 were more probable in fractured samples. JAG1 did not interact with Notch receptors in intact samples (Supplemental Figure 2B).

### Effect of Notch1 overexpression on fracture healing.

**Figure 3:**
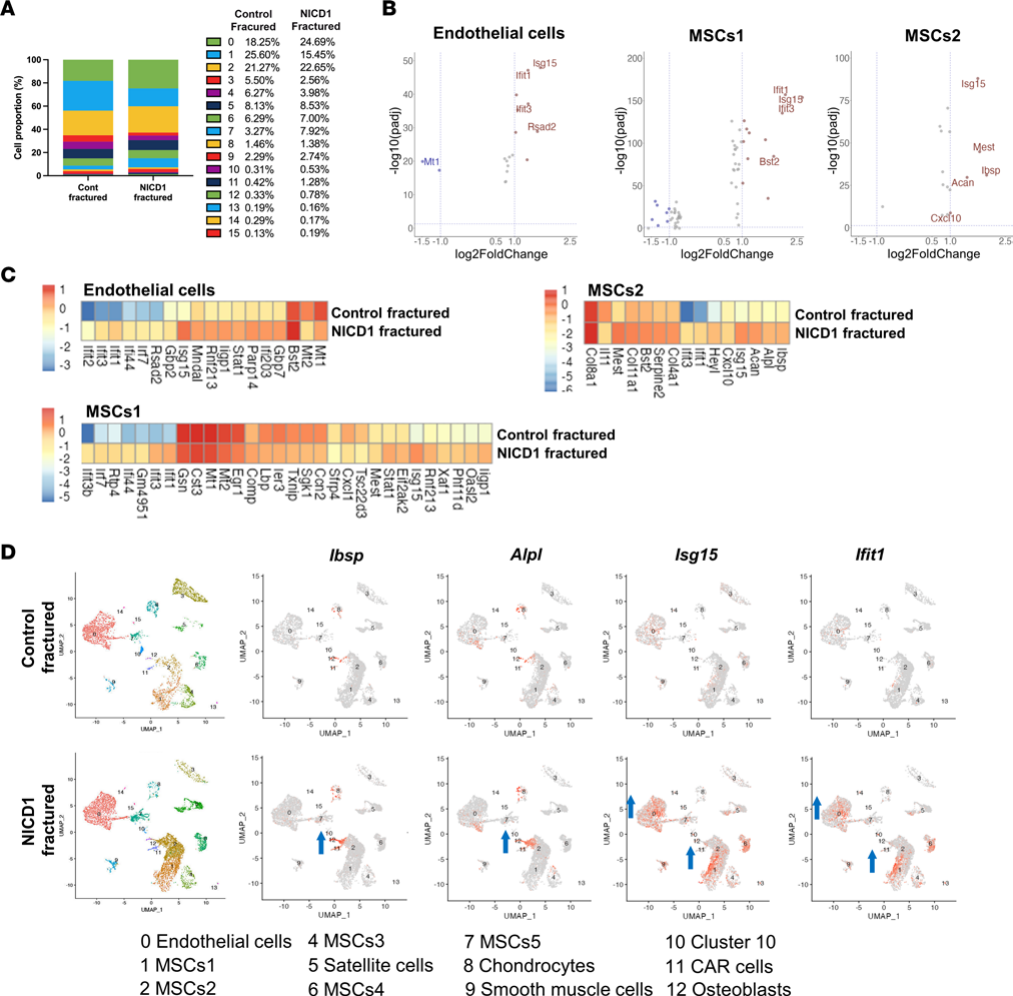
Overexpression of NICD1, during fracture healing, induces osteogenic and IFN signaling gene expression. (**A**) Proportion of cells within each cluster in control Cre^–^ and Cre^+^ αSMACreER/NICD1 fractured samples. (**B**) Volcano plots showing differentially expressed genes in NICD1 fractured periosteal samples compared to NICD1 intact samples of EC, MSCs1, and MSCs2 clusters. (**C**) Heatmaps with the complete list of differentially expressed genes. Color intensity represents the mean gene expression of all cells within the cluster. (**D**) Feature plots of αSMACreER/NICD1 fractured Cre^–^ and Cre^+^ samples showing increased osteogenic genes (*Ibsp*, *Alpl*) and IFN signaling genes (*Isg15*, *Ifit1*).

To address the role of Notch1 signaling in the early regulation of bone healing, we isolated intact and fractured periosteal cells (3 dpf) from αSMACre^+^/NICD1 and Cre^–^/NICD1 mice. We observed an increase in the proportion of ECs (cluster 0) and some MSCs in clusters 6 (MSCs4) and 7 (MSCs5) within fractured samples with Notch1 overexpression (Figure 3A). scRNA-seq data indicated that fracture (Cre^–^ and Cre^+^ samples) increases the proportion of mesenchymal populations, with a decreased proportion of ECs in both intact Cre^–^ and Cre^+^ samples (Supplemental Figure 3). We postulate that this is not due to reduction of EC number, but due to a greater increase in MSC proliferation. The MSCs2 cluster from the injured periosteum of NICD1-overexpressing animals (Cre^+^) exhibited significantly increased expression of *Ibsp* and *Alpl* compared with the injured Cre^–^ callus (Figure 3, C and D). Overexpression of NICD1 in fractured samples led to increased expression of genes involved in IFN signaling (i.e., *Ifit1*, *Isg15*, *Ifit3*, *Iigp1*, *Ifi203*, and *Ifi27l2a*) in MSC and EC clusters (Figure 3, B–D).

Despite the high proliferative activity of mesenchymal populations at 3 dpf, CD45^+^ hematopoietic cells remained a dominant population (~88% CD45^+^ cells). RNA-seq analysis revealed the presence of primarily myeloid cells (neutrophils, macrophages, and dendritic cells), with a small proportion of lymphocytes (Supplemental Figure 4). Neutrophils and macrophages expressed significantly higher levels of *Tnf* and *Il1b* upon fracture (Supplemental Figure 4C). However, overexpression of NICD1 in the αSMA-expressing cells resulted in a decrease in proinflammatory signals in CD45^+^ cells (*Il1b*, *Tnf*, and *Il6*) after bone injury, confirming interactions between MSCs and hematopoietic cells (Supplemental Figure 4D).

### EC-derived DLL4 as a regulator of bone healing.

We confirmed the expression of *Dll4* and *Jag2* in ECs, while *Jag1* was expressed in MSCs (Supplemental Figure 5). We did not detect expression of *Dll1* or *Dll3* in periosteal populations. *Notch1* showed strong expression in ECs, committed mesenchymal progenitors, CAR cells, satellite cells, and smooth muscle cells, while a low level of *Notch2* was present in all nonhematopoietic cells within the callus (Supplemental Figure 5). *Hes1*, a downstream signaling gene of Notch, exhibited the highest expression in periosteal cells.

**Figure 4:**
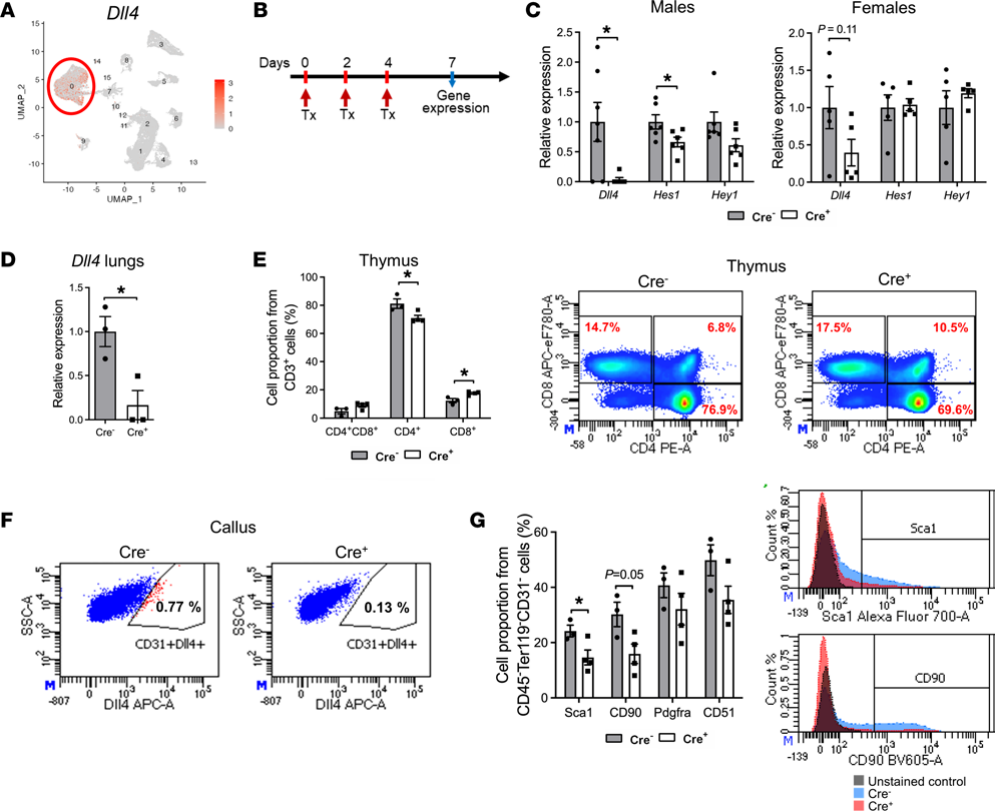
Notch signaling in fracture healing. (**A**) Feature plot of *Dll4* expression within periosteal cells. (**B**) Experimental design of recombination efficiency evaluation by determining mRNA expression of *Dll4* and Notch downstream signaling genes (*Hes1* and *Hey1*) in male and female mice. To induce recombination, tamoxifen (Tx) was injected at 0, 2, and 4 dpf, and gene expression was evaluated at 7 dpf. *Dll4* was successfully deleted in male mice, with decreased expression of *Hes1* and *Hey1* in the Cre^+^ callus (**C**) and lungs (**D**). Males: Cre^–^ *n* = 6, Cre^+^ *n* = 6; females: Cre^–^ *n* = 5, Cre^+^ *n* = 5. (**E**) Proportion of thymocyte subpopulations in the thymus, where deletion of *Dll4* in ECs on day 5 after the first tamoxifen injection induced a small decrease in CD4^+^ cells and an increase in CD8^+^ cells, with representative dot plots. (**F**) Flow cytometry analysis showing dot plots of CD31^+^ cells within the callus at 5 dpf expressing DLL4. (**G**) Proportion of callus cells expressing Sca1, CD90, PDGFRα, and CD51 at 5 dpf, with representative overlaid histograms of Sca1 and CD90 cell expression (unstained control, Cre^–^ and Cre^+^ sample). In **E**–**G**, Cre^–^ *n* = 3, Cre^+^ *n* = 4. Unpaired, 2-tailed Student’s *t* test. *P < 0.05.

The only population expressing *Dll4* was endothelial (Figure 4A), prompting us to pursue experiments disrupting *Dll4* (Dll4Δ) in ECs using Cdh5CreER. We performed a lineage tracing experiment of Cdh5 cells using Cdh5CreER/Ai9 (Supplemental Figure 7). We injected tamoxifen on the day of fracture. Cdh5/Ai9 cells are ECs positive for the CD31 endothelial marker (Supplemental Figure 7). Lineage tracing of fractured femurs revealed already-formed blood vessels with Cdh5Cre/Ai9-labeled ECs in the bone marrow and in the muscle surrounding the fracture (Supplemental Figure 6a′). Cdh5/Ai9 cells were present within the expanded periosteum at 3 dpf (Supplemental Figure 6a′′). We observed a lack of infiltrating blood vessels within the cartilaginous tissue on days 7 and 10 (Supplemental Figure 6, b′ and C), but abundant ECs were present within the mineralized callus (Supplemental Figure 6, D and E). At later time points of fracture healing, blood vessels surrounded the callus tissue and penetrated the remodeling callus (Supplemental Figure 6e′).

We evaluated fracture healing in young adult (10- to 12-week-old) mice lacking *Dll4* in ECs using Cdh5CreER. We induced Cre activity by intraperitoneal injection of tamoxifen (75 mg/kg) on 0, 2, and 4 dpf (Figure 4B). Real-time PCR confirmed efficient *Dll4* deletion in callus samples collected 7 dpf from male Cre^+^ mice compared with Cre^–^ mice (Figure 4C) and in lung tissue abundant in blood vessels (Figure 4D). This deletion of *Dll4* within ECs led to a decrease in expression of *Hes1* in the bulk calluses of Cre^+^ mice (Figure 4C). Notably, deletion of *Dll4* was less efficient in female mice (not statistically significant), and the *Hes1* and *Hey1* genes were not downregulated in female animals (Figure 4C).

Although Notch signaling is crucial for T lymphocyte differentiation (28), our inducible model of *Dll4* deletion with tamoxifen did not lead to differences in B220^+^ and CD3^+^, CD4^+^, and CD8^+^ proportions within the bone marrow of an intact bone at 5 dpf (Supplemental Figure 8, A and B). We observed differences in differentiating T cell populations (CD4^+^CD8^+^, CD4^+^, and CD8^+^ T cells) within the thymus, where deletion of *Dll4* decreased the proportion of CD4^+^ cells and increased CD8^+^ cells (Figure 4E).

We further evaluated the phenotype of the MSCs within the callus tissue at 5 dpf. With *Dll4* deletion within ECs, the proportion of nonhematopoietic (CD45^–^Ter119^–^) cells was significantly decreased (23.3% fewer CD45^–^Ter119^–^ cells, with 40.6% in Cre^+^ vs. 31.2% Cre^–^ animals; *P* < 0.01). The proportion of ECs (CD45^–^CD31^+^) within the groups was unchanged (4.30% ± 0.91% in Cre^+^ and 5.47% ± 1.11% in Cre^–^). The lack of DLL4 expression in CD45^–^CD31^+^ cells was confirmed by flow cytometry (Figure 4F). The MSC population (CD45^–^Ter119^–^CD31^–^) within the callus of Cre^+^ mice exhibited a significantly decreased proportion of Sca1^+^ cells (*P* < 0.05) and a lower proportion of CD90^+^ cells (*P* = 0.05) (Figure 4G). The Sca1^+^CD51^+^ population was also decreased in Cre^+^ animals compared with Cre^–^ littermate controls in the periosteal callus at 5 dpf (Supplemental Figure 8C).

**Figure 5:**
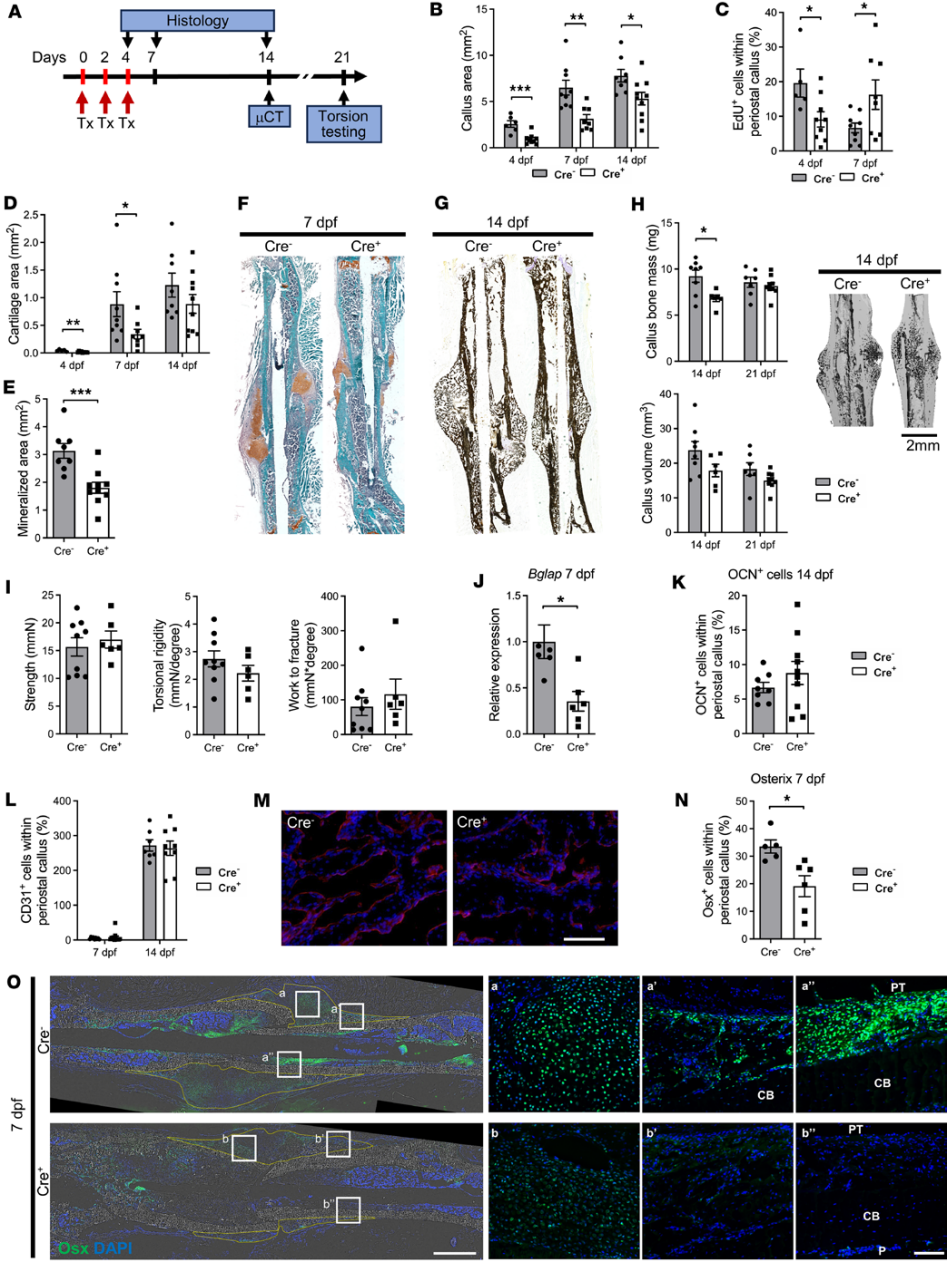
Deletion of *Dll4* in ECs impairs fracture healing. (**A**) Experimental design. Deletion of *Dll4* in ECs was induced by injecting tamoxifen (Tx) at 0, 2, and 4 dpf. Fractured bone samples were evaluated on day 4, 7, and 14 by histology and day 21 by microCT and torsion testing. (**B**) *Dll4* deletion led to a decreased callus size at 4 dpf (Cre^–^ *n* = 6, Cre^+^ *n* = 9), 7 dpf (Cre^–^ *n* = 9, Cre^+^ *n* = 8), and 14 dpf (Cre^–^ *n* = 8, Cre^+^ *n* = 10). (**C**) *Dll4* deletion also resulted in decreased proliferation at 4 dpf (Cre^–^ *n* = 5, Cre^+^ *n* = 9), which increased by 7 dpf (Cre^–^ *n* = 9, Cre^+^ *n* = 8) in Cre^+^ compared with Cre^–^ animals. (**D** and **F**) Cre^+^ mice had significantly less cartilage. Sample numbers at 4 dpf (Cre^–^ *n* = 5, Cre^+^ *n* = 9), 7 dpf (Cre^–^ *n* = 9, Cre^+^ *n* = 8), and 14 dpf (Cre^–^ *n* = 8, Cre^+^ *n* = 10) were analyzed by evaluating Safranin O–stained sections, as observed on representative sections. (**E** and **G**) Mineralized area was analyzed by von Kossa staining (Cre^–^ *n* = 8, Cre^+^ *n* = 10), as shown on Cre^-^ and Cre^+^ representative sections. (**H**) MicroCT analysis showed decreased callus bone mass on day 14 and no difference in callus volume, with representative 3D reconstructions of Cre^–^ and Cre^+^ fractures on the right. At 7 dpf, Cre^–^ *n* = 8, Cre^+^ *n* = 6; and at 21 dpf Cre^–^ *n* = 7, Cre^+^ *n* = 9. (**I**) Biomechanical properties were evaluated by torsion testing and are presented as bone strength (maximum torque), stiffness as a measure of torsional rigidity, and toughness as a work to fracture measure, with no change between Cre^–^ and Cre^+^ fractured bones at 21 dpf. Cre^–^ *n* = 9, Cre^+^ *n* = 6. (**J**) *Bglap* gene expression analysis at 7 dpf (Cre^–^ *n* = 6, Cre^+^ *n* = 6). (**K**) Histological analysis of osteocalcin-stained samples at 14 dpf (Cre^–^ *n* = 8, Cre^+^ *n* = 10). (**L**) Evaluation of CD31^+^ cells within the periosteal callus at 7 dpf (Cre^–^ *n* = 9, Cre^+^ *n* = 12) and 14 dpf (Cre^–^ *n* = 7, Cre^+^ *n* = 9). Deletion of *Dll4* in ECs with (**M**) representative magnified images of CD31 staining within the mineralized callus at 14 dpf. Scale bar: 200 μm. (**N**) Proportion of osterix-stained cells within the periosteal callus (Cre^–^ *n* = 5, Cre^+^ *n* = 6). (**O**) Representative images of osterix-stained fractured femurs with magnified areas of cartilaginous callus (a and b), mineralized callus (a’ and b’), and cortical bone with the area next to the pin insertion (a” and b”). The analyzed periosteal callus is shown by the yellow line. Scale bars: 1 mm (left) and 100 μm (right). PT, pin trace; CB, cortical bone; P, periosteum. Unpaired, 2-tailed Student’s *t* test. *P < 0.05, **P < 0.01.

We evaluated how the deletion of *Dll4* in ECs affects callus healing through histology, micro-computed tomography *(*microCT), and mechanical testing. Histological evaluation of femur fractures revealed a smaller callus area at 4, 7, and 14 dpf (Figure 5, A and B). The reduced callus area indicated lower proliferation under DLL4 deficiency, which was confirmed by a significant decrease in EdU^+^ cells within the callus area of Cre^+^ animals at 4 dpf (Figure 5C). However, at 7 dpf, Cre^+^ animals exhibited more EdU^+^ cells. Despite the increased proliferation later in the healing process, the callus area did not reach the size of their littermate control Cre^–^ animals, indicating a phenotype of delayed healing. We observed less cartilage area in Cre^+^ animals at 4 and 7 dpf (Figure 5, D and F), and significantly less mineralized tissue at 14 dpf (Figure 5, E and G). MicroCT analysis confirmed decreased bone mass within the callus at 14 dpf (Figure 5H). By day 21, there was no difference in bone mass and callus volume (Figure 5H), nor any difference in the biomechanical properties of the fractured femurs (Figure 5I). Although we observed decreased expression of *Bglap* in Cre^+^ mice within the callus at 7 dpf (Figure 5J), histological analysis of osteocalcin on 14 dpf did not show any difference in osteocalcin^+^ cells within the callus (Figure 5K).

Given the important role of DLL4 in vasculogenesis, we evaluated the presence of autocrine effects of *Dll4* deletion on EC number within the callus. We stained femur fracture sections at 7 and 14 dpf for the EC marker CD31 (Figure 5, L and M), which colabels Cdh5/Ai9 cells within the callus area (Supplemental Figure 7). We observed no difference in CD31^+^DAPI^+^ cells within the periosteal callus when comparing the Cre^–^ and Cre^+^ groups (Figure 5, L and M). Next, we evaluated the effects on the population of osteoprogenitors and determined a 43% decrease in osterix^+^ (Osx^+^) cells in the periosteal callus upon deletion of *Dll4* in ECs (33.4% in Cre^–^ compared with 19.1% in Cre^+^) (Figure 5, N and O).

As an important control, we also crossed the *Dll4*-floxed mice with αSMACreER to verify the EC-specific requirements for DLL4 in fracture healing. We evaluated the expression of *Dll4* in the fractured callus at 7 dpf upon tamoxifen treatment (at 0, 2, and 4 dpf) in both Cre^–^ and Cre^+^ animals. We observed no change in *Dll4* expression when *Dll4* was deleted under the αSMA promotor using αSMACreER/Dll4Δ mice (Supplemental Figure 9), and no effect on *Hes1* and *Hey1* expression, which is consistent with our scRNA-seq data showing the lack of *Dll4* expression in all mesenchymal clusters (Supplemental Figure 5).

### Notch ligands as a therapeutic approach for bone healing.

**Figure 6:**
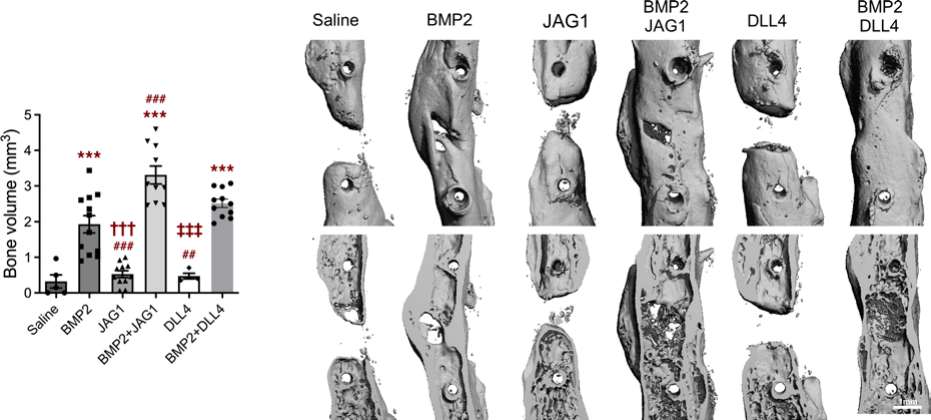
Notch ligands improve critical size femoral defect healing. BMP2 (5 μg), JAG1 (6 μg), and DLL4 (5 μg) alone or in combination were applied to a collagen scaffold (Medtronic). Femurs were evaluated by microCT and bone volume within the defect area was determined. JAG1 in combination with BMP2 resulted in significantly more bone within the defect compared with BMP2 only, with cortical bone formation in the whole length of a defect. On the right, representative 3D reconstructions of healing femoral defects 9 weeks after defect surgery. Saline, *n* = 5; BMP2, *n* = 12; JAG1, *n* = 11; BMP2 + JAG1, *n* = 10; DLL4, *n* = 4; BMP2 + DLL4, *n* = 11. One-way ANOVA with Bonferroni’s post hoc test. ***P < 0.001, statistically different from saline; ^##^*P* < 0.01, ^###^*P* < 0.001, statistically different from BMP2; ^†††^*P* < 0.001, statistically different from BMP2 + JAG1; ^‡‡‡^*P* < 0.001, statistically different from BMP2 + DLL4.

We used a clinically relevant critical size femoral defect model to examine the role of Notch signaling during bone healing. We utilized DLL4 and JAG1 alone or in combination with BMP2. Because *Jag1* is more broadly expressed within periosteal tissue (MSCs, muscle cells, preosteoblasts, and ECs) than *Dll4* (Supplemental Figure 5), we expected it to exhibit a stronger healing phenotype with JAG1 treatment. JAG1 (6 μg) alone was not sufficient to induce bone bridging of a 2 mm defect within 9 weeks of healing. However, when combined with 5 μg of BMP2, JAG1 significantly increased bone volume within the defect area compared with BMP2 treatment alone (Figure 6). Similarly to JAG1, DLL4 treatment (5 μg) alone did not result in bridging of a defect. However, when combined with BMP2, bridging of the defect was achieved. The cortical bone morphology within the defect resembled physiological appearance more than with BMP2 treatment alone.

## Discussion

An analysis of periosteal cells from intact and fractured bones during early fracture healing using scRNA-seq confirmed an increase in early mesenchymal stem/progenitor cells (MSCs1 and MSCs2) and their transition into mature skeletal lineages, including chondrocytes and osteoblasts. The MSCs1 population, characterized by low proliferative potential and inhibition of genes involved in the cell cycle, exhibited an increased proportion of replicating cells in the G_2_M phase following fracture. A trajectory analysis revealed the transition of MSCs1 into MSCs2 with the dramatic changes of the MSCs1 population expressing *Clec3b*, a marker of early stem/progenitor cells, transitioning into a highly proliferative MSCs2 population characterized by the expression of *Acta2*. Following fracture, the MSCs1 population expanded, showing a decrease in *Clec3b* expression and an increase in the expression of periosteal progenitor markers such as *Acta2*, periostin, and *Cxcl12*. A similar transition from *Clec3b*-expressing early mesenchymal progenitor cells to a population expressing *Postn*, indicative of a more mature state, has been observed in bone marrow MSC lineages (29). The expression of chemokines (*Cxcl1* and *Cxcl5*) within MSCs was increased upon fracture, which facilitates the mobilization of macrophages and neutrophils to the fracture site (30). Additionally, we observed an increase in the inflammatory mediator *Ptx3*, a pattern recognition molecule with a role in matrix remodeling. Research indicates that fracture healing in *Ptx3*-knockout mice is partially impaired due to reduced osteoblast function (31).

We extend our previous work on Notch signaling in fracture healing, in which we demonstrated that Notch signaling promotes bone healing. We reported that efficiency of αSMACreER is approximately 50%–60% (32, 33), and we were able to induce overexpression of NICD1 using the same promotor (22). In this study, we aimed to identify novel regulatory pathways through which Notch ligands and signaling via NICD1 impact healing. We confirmed increased induction of osteogenesis in fractured samples with NICD1 overexpression in αSMA^+^ osteoprogenitor cells and observed decreased expression of proinflammatory genes (*Tnf* and *Il6*) 3 days after the fracture. These results are consistent with previously published data showing that systemically impaired canonical Notch signaling during fracture healing leads to impaired healing, characterized by increased neutrophil infiltration in the callus area and elevated *Tnf* and *Il1b* expression (34). Although the initial inflammatory response is important to begin the healing process, achieving physiological healing requires a delicate balance between pro- and antiinflammatory signals (35, 36). Our data indicate that an earlier decrease in proinflammatory signals during bone regeneration might be beneficial for the healing process (Supplemental Figure 4D). Interestingly, the increase in NICD1 in MSCs not only affected proinflammatory cytokines, but also resulted in the induction of multiple IFN pathway molecules, such as *Isg15*, *Ifit1*, *Ifit3*, *Iigp1*, *Ifi203*, and *Ifi27l2a*, in MSCs and ECs within the callus. *Isg15* was the most significantly increased gene in the MSCs1 cluster within the fractured NICD1 sample. Studies have shown that *Isg15* deficiency leads to decreased trabecular bone volume, as well as a reduced bone formation rate, femoral cortical bone thickness, and biomechanical properties (37). Notch activation in helper T cells can regulate *Ifng* expression at the *Ifng* CNS-22 enhancer (38). Furthermore, the presence of IFN-γ at the fracture site can induce IFN downstream signaling genes within ECs and MSCs in the fracture.

We next sought to understand the specific role of Notch ligands during bone healing. Notch receptors and ligands are expressed throughout all stages of bone healing (15). We observed that *Dll4* and *Jag2* are the only Notch ligands expressed by ECs, with *Dll4* expression being restricted to ECs. Notably, global *Dll4* knockout results in embryonic lethality due to improper blood vessel formation, and *Dll4^–/–^* embryos show abnormal accumulation of ECs in the apical portion of intersomitic vessels (39).

The periosteum, a highly vascularized tissue, is required for normal bone healing. However, a signaling role for blood vessel ECs in fracture healing has never been demonstrated to our knowledge. Thus, we investigated the EC-specific role of DLL4 using Cdh5CreER mice. We selected Tg(Cdh5Cre/ER)^1Rha^ for its specificity in targeting ECs (40) and lack of Cre expression in hematopoietic cells or other non-endothelial tissue (41). We deleted *Dll4* in both EC (Cdh5CreER) and MSC (αSMACreER) lineages. As expected, deletion of *Dll4* in MSCs did not lead to a decrease in total *Dll4* within the callus tissue, as *Dll4* is not expressed in mesenchymal populations. *Dll4* deletion in ECs using Cdh5CreER mice showed a lack of *Dll4* expression in the callus and lungs, accompanied by a significant decrease in expression of a Notch downstream signaling gene (*Hes1*) in the callus of male animals. The lack of *Dll4* decreased the proportion of early SSPCs, resulting in a lower proportion of Sca1^+^ cells and a tendency to decrease CD90^+^ cells as well. Additionally, Sca1^+^CD51^+^ cells were significantly reduced with the deletion of *Dll4* compared with the Cre^–^ control. Studies have shown that Notch signaling affects periosteal progenitors in the healing process (22, 42). Inhibiting Notch1 with an antibody decreased the proportion of αSMA lineage–traced osteoprogenitors. By contrast, NICD1 overexpression in osteoprogenitors increased αSMA lineage–traced osteoprogenitors and mature osteocalcin^+^ osteoblasts within the fracture, as well as increased the presence of CD51^+^ and CD34^hi^ cells and Sca1^+^CD51^+^ cells in a periosteal scratch injury (22, 42). This early activation of Notch signaling in bone regeneration has been shown to induce proliferation and differentiation of early osteoprogenitor cells, which has a beneficial effect on bone regeneration (22, 43, 44).

Besides its role in fracture healing, Notch signaling activation is crucial for T cell differentiation. The decision of common lymphoid progenitors to differentiate into T or B cells is based on Notch signaling, which induces T-lineage commitment within the thymus. Thymic epithelial cells abundantly express Notch ligands and coordinate T cell differentiation (45). When *Dll4* is deleted in thymic epithelial cells, decreased Notch signaling has been observed within the thymus, leading to the accumulation of CD4^–^CD8^–^ double-negative cells and aberrant B cell accumulation within the thymus (46). Our data showed that deletion of *Dll4* in ECs did not influence the proportion of T and B cells within the bone marrow. However, the lack of DLL4 led to a small but significant decrease in CD4^+^ cells and an increase in the proportion of CD8^+^ cells within the thymus. The impact of different T cell populations on fracture healing needs to be further determined and will be the focus of our future research.

Studies have demonstrated the importance of endothelial Notch signaling in coupling vasculogenesis and osteogenesis. Deletion of Rbpjκ results in decreased CD31^hi^Emcn^hi^ vessels, leading to decreased bone size (47). Deletion of *Dll4* in ECs recapitulated this phenotype, resulting in decreased bone size, less trabecular bone, accumulation of Osx^+^ cells, and a reduction in Runx2^+^ early osteoprogenitors (47). In our study targeting *Dll4* deletion in ECs during early fracture healing, we did not observe an effect on the number of ECs within the callus. However, the lack of *Dll4* led to a significant decrease in periosteal SSPC proliferation, resulting in smaller callus tissue, likely due to a decrease in mesenchymal progenitor expansion (fewer Sca1^+^, CD90^+^, and Osx^+^ cells). The smaller callus size was accompanied by less bony callus on day 14 in Cre^+^ animals. However, by day 21, the differences in bone volume and bone mass did not persist, and there were no differences in the biomechanical properties of the fractured bone. This result indicates that DLL4 is an important mediator involved in early bone healing, with potential compensation later in the healing process from other ligands, such as JAG1, which is expressed in different MSCs and smooth muscle cells in callus tissue. Indeed, a study found that JAG1 expression in MSCs is required for intramembranous bone formation in a marrow ablation model (23). Our data indicate that fracture healing is delayed with *Dll4* deletion in ECs.

Interestingly, we did not observe significant deletion of *Dll4* in Cdh5CreER/DllΔ female mice. We previously reported lower efficiency of Cre recombination in female animals using the αSMA promoter (22, 23, 48). Sex differences and/or decreased targeted gene expression in females could lead to inefficient Cre recombination (23). Potentially, increasing the tamoxifen dose could be one solution to increase Cre recombination; however, research has shown that higher doses of tamoxifen are toxic (49, 50).

We determined the potential therapeutical effects of Notch ligands during critical femoral defect healing. Critical size bone defects do not heal spontaneously without additional interventions, such as treatment with BMP2, BMP7, and PDGF. A high dose must be used to achieve osteogenesis but can result in significant side effects. Although soluble Notch ligands can act as a Notch signaling antagonists (51), when they are on the cell surface, bound to a tissue culture plate or scaffold, they have the ability to act as agonists and activate Notch signaling (23, 44, 52, 53). Studies have shown that a bone-targeting, high-affinity version of the soluble ligand DLL4 can induce significant bone formation in male mice (52). DLL4 actions on critical size bone defect healing have not been studied thus far. When we applied DLL4 or JAG1 alone on the scaffold to affect critical size femoral defect healing, bone formation was not sufficient to bridge the defect gap. We showed that DLL4 can complement BMP2-induced osteogenesis and bridge the gap of a defect. We also tested whether JAG1, as a ligand expressed in MSCs and smooth muscle cells, would have a better bridging effect since more cells express it. JAG1 applied in combination with BMP2 bridged the defect and significantly increased bone volume in the defect compared with BMP2-only treatment. Previous studies have demonstrated the benefits of applying recombinant JAG1 to the scaffold in intramembranous healing of rat calvarial defects, where JAG1 treatment can lead to more physiological healing without bone overproduction, as observed with BMP2 (23).

The dose of BMP2 that we used was chosen because it is a more moderate level than that often used in comparable studies and was chosen so that we would still be able to see the coincident impact of BMP2 and Notch ligand delivery. Although the combined impact of dual factor delivery is “mild,” the fact of the matter is that the co-delivery of BMP2 and JAG1 increases bone formation more than 50% than BMP2 alone. From a clinical perspective, if doses of BMP2 can be reduced in humans, through the coactivation of Notch signaling, then this could provide an approach for faster and more robust healing without the deleterious impacts of supraphysiological bone formation. Our data show that there is no bone outgrow and no ectopic bone formation, which at this level seems better than what clinical cases are showing.

We confirmed the importance of Notch signaling in osteoprogenitors, which led to distinct transcriptional changes in SSPCs and their lineages, as well as hematopoietic cells during the healing process. Deleting *Dll4* in ECs impaired the healing process, suggesting that early Notch signaling through endothelial *Dll4* expression is required for proper healing initiation (resulting in a larger callus with more cartilage) and potentially osteoprogenitor differentiation (increased *Osx* in Cre^–^ animals) in the fracture healing process. Additionally, combining BMP2 with the Notch ligand DLL4 or JAG1 showed potential therapeutical benefits, resulting in improved bridging of critical size femoral defects with activation of Notch signaling.

## Methods

### Sex as a biological variable.

All the experiments were conducted on male animals and some experiments were performed in males and females, as reported in the main text.

### Mice.

The following mouse lines were used: αSMACreER (2), Cdh5CreER (sourced from Guo-Hua Fong, UConn Health) (40), Ai9 (JAX, 007909), *Gt(ROSA)26Sor^tm1(Notch1)Dam/J^* (JAX, 008159), and DLL4^flox^ (sourced from Ivan Maillard, University of Pennsylvania) (54). The scRNA-seq analysis utilized 4-month-old mice, whereas the critical size femoral defects were performed in adult 3.5- to 7-month-old mice. The mice were group-housed in ventilated cages within our animal facility and kept at 22°C (±1°C) with 50% relative humidity, a 12-hour light/dark cycle, and ad libitum access to water and food (Teklad T.2918.15, Envigo). Cdh5CreER were kept on breeding diet (Teklad T.2919.10, Envigo). For inducible Cre activation, animals received intraperitoneal injections of 75 mg/kg of tamoxifen as outlined in the experimental design. Cre^+^ and Cre^–^ littermate control mice were injected to control for the effect of tamoxifen on the bone phenotype.

### Genotyping.

DNA was extracted from tail tissue biopsies and Cre and NICD1 genotyping was performed as previously described (22, 55). Genotyping for *Dll4* was performed using 5′-GTGCTGGGACTGTAGCCACT-3′ forward and 5′-TGTTAGGGATGTCGCTCTCC-3′ reverse primer (54).

### Fracture model.

Fractures were performed in αSMACreER/NICD1 and Cdh5CreER/Dll4Δ Cre^+^ and their littermate control Cre^–^ mice. Transversal femoral fractures were made using previously described methods (22). To confirm fracture placement and the healing process, x-ray (Parameter 2D, Kubtec) was used. Destabilized fracture evidenced by pin displacement, absence of a fracture callus by day 14, multifracture, and loss of movement of the limb were exclusion criteria. Buprenorphine HCl (0.08 mg/kg, Reckitt Benckiser Pharmaceuticals) was administrated as analgesia subcutaneously every 10–12 hours for 48 hours following the time of fracture.

### scRNA-seq.

scRNA-seq was performed using intact and fractured periosteal cells (3 dpf) from αSMACre^+^/NICD1 and Cre^–^/NICD1 mice. To induce NICD1 overexpression, Cre^–^ and Cre^+^ mice were injected with tamoxifen (75 mg/kg) on the day of fracture and 2 dpf (or if intact, 3 and 1 day before sacrificing animals). Three animals from each group were sacrificed, and periosteum (intact) or periosteal callus (fractured) samples were pooled and digested as previously described (22, 55) using Collagenase P (Roche) and hyaluronidase (Sigma-Aldrich) for 1 hour at 37°C with shaking. Live MSCs (CD45^–^) and hematopoietic cells (CD45^+^) were sorted, and scRNA-seq was performed (10× Genomics). Periosteal cells were digested and stained for CD45 and Ter119 (see Supplemental Table 2), and with propidium iodide to exclude dead cells. CD45^–^Ter119^–^ and CD45^+^Ter119^+^ cells were sorted (BD FACSymphony S6 Cell Sorter) and 12,000 live cells were loaded into a 10× Chromium X controller with approximately 10,000 cells barcoded for scRNA-seq using a 10× Genomics Chromium Single Cell 3′ Kit (v3.1 NEXTGEM, CG000315). Libraries were sequenced on a NovaSeq 6000 S4 flow cell (200 cycle v1.5, Illumina), with an average sequencing depth of 100,000 reads per cell for CD45^–^ and 50,000 reads per cell for CD45^+^. For FASTQ generation and alignment, Illumina basecall files were converted to FASTQs using bcl2fastq v2.20.0.422 (Illumina) and FASTQ files associated with the gene expression libraries were aligned to the mm10 genome using the Cell Ranger count pipeline (v6.1.2; 10× Genomics). Data analysis was performed in Rstudio using the Seurat package (v4.3.0; https://satijalab.org/seurat/articles/get_started.html), where the functions FindIntegrationAnchors and IntegrateData were used to integrate data. Principal component analysis was performed, and clusters were identified using 40 principal components at a resolution of 0.25 for CD45^–^ cells and 0.4 for CD45^+^ cells. The cell clusters were visualized using uniform manifold approximation and projection (UMAP) plots. Cell-type clusters were determined based on cluster-specific marker genes.

### Gene expression analysis.

To determine Cre efficiency in deleting *Dll4*, we used real-time PCR to measure *Dll4* gene expression from callus samples at 7 dpf. RNA was isolated using TRIzol reagent (Thermo Fisher Scientific), as we described previously (56), purified from DNA by DNase treatment (Invitrogen), followed by synthesis of 1 μg of RNA into cDNA by ImProm II (Promega). TaqMan probes for *Dll4* (Mm04238539_m1), *Hes1* (Mm01342805_m1), *Hey1* (Mm00468865_m1), *Bglap* (Mm03413826_mH), and *Gapdh* (Mm99999915_g1) with TaqMan Universal Mastermix (Thermo Fisher Scientific) were used for analyzing gene expression. Data were normalized to the *Gapdh* housekeeping gene, calculated using 2^–ΔΔCt^, and presented as fold change in the Cre^+^ compared with the Cre^–^ group.

### Critical size femoral defects.

C57BL/6J animals aged 3.5–7 months were used to evaluate critical size femoral healing (2 mm) using an established protocol and commercially available external fixation (MouseExFix, RISystem AG) (48). DLL4 (5 μg, R&D Systems), JAG1 (6 μg, R&D Systems), or their combination with BMP2 (5 μg) was applied on an absorbable collagen sponge as a scaffold (Medtronic) cut to size (3 × 4 × 4 mm) onto which growth factors were loaded in a 7 μL volume. Male mice were used, as their larger femur size allows for the defect and appliance placement. Medicated water (1.5–4.5 mg/mouse/day sulfamethoxazole/trimethoprim; Aurobindo Pharma USA, Inc.) was given to animals undergoing surgery (2 days before and 2 weeks after) to prevent infection.

### Flow cytometry.

Callus and bone marrow cells were collected and digested as described previously (22). Thymocytes were collected by teasing apart the thymus between the frosted ends of 2 microscope slides. Samples were incubated with the mixture of antibodies for 30 minutes on ice, washed, and DAPI was added before sample acquisition to evaluate only live cells. Cell acquisition was performed using an LSR II (BD Biosciences), and cell analysis was conducted using Diva 8 software. Unstained samples, single-stain controls, and fluorescence-minus-one controls were used for setting gates. A list of antibodies is provided in Supplemental Table 2.

### Histology.

Fractured femurs were fixed in 4% paraformaldehyde (PFA) for 3 days at 4°C, followed by overnight incubation in 30% sucrose/PBS. Pins were removed and fractured femurs were embedded in cryomatrix (Thermo Fisher Scientific). Sections (7 μm thick) were cut using a tape transfer system (Section-lab). We evaluated proliferation by EdU incorporation (Click-iT EdU Alexa Fluor 647 Flow Cytometry Assay Kit, Molecular Probes), callus area, and cartilage area within the callus by Safranin O. Mineralization was determined by von Kossa staining, and CD31 staining as previously described (22, 57).

Two sections per sample were collected from the middle of a fracture or bone sample after critical size defects, stained, and scanned using an Axioscan 7 microscope (Zeiss). For automated cell counting, we used ImageJ (NIH) as previously published (48).

### MicroCT and mechanical testing.

After fractured femurs were collected for microCT and torsion testing at 3 weeks after fracture, they were dissected, soaked in PBS, wrapped in gauze, and stored at –20°C until analysis. Two weeks after fracture when samples were collected for microCT and histology, femurs were fixed in 4% PFA for 3 days and transferred to PBS for microCT. After microCT scans were performed, samples were incubated in 30% sucrose and evaluated by histology as described above. For microCT, a μCT50 (Scanco Medical AG) with a voxel size of 16 μm, 55 kV, and intensity of 145 μA was used to analyze 200 sections from the middle of the fracture. To determine the biomechanical properties of the fractured bone, we performed torsion testing using an Electroforce (ELF) 3220 Series III (TA Instruments) mechanical testing machine with axial and rotational actuators and a 225N/6.64 Nm load/torque cell, with a data acquisition rate of 10 Hz and 1 degree/s torsion, as described previously (22).

Femoral defects were analyzed by microCT (μCT50), and bone healing ability was evaluated 9 weeks after defect surgery. We scanned defects with a 16 μm voxel size, at 55 kV, 145 μA, and 500 ms integration time, and bone volume was determined within the defect. Segmentation of bone was performed using a constrained Gaussian filter to reduce noise, applying a standardized threshold of 220 (per 1000).

### Statistics.

All data are presented as mean value ± standard error of the mean (SEM). To perform statistical analysis, GraphPad Prism 10 software was used. An unpaired, 2-tailed Student’s *t* test was performed to determine differences between 2 groups, with *P* less than 0.05 set as the threshold for statistical significance between the tested groups. When multiple groups were analyzed, 1-way ANOVA was used, followed by Bonferroni’s post hoc test. The number of bone samples included in study is listed in the figure legends. Samples were only pooled for scRNA-seq analysis.

### Study approval.

All animal procedures were approved by the UConn Health Institutional Animal Care and Use Committee and performed in a facility accredited by the Association for Assessment and Accreditation of Laboratory Animal Care.

### Availability of data, materials, and methods.

All supporting data are provided in the Supporting Data Values file or will be provided by the corresponding author upon request. The processed scRNA-seq files and associated metadata have been deposited in the NCBI Gene Expression Omnibus (GEO) database (GSE260749).

## Author contributions

IK, SN, and HT performed experiments, data acquisition, analysis, interpretation of data, and wrote the manuscript. SHR, VS, TAS, and KDH performed experiments and data analysis. IK and SN conceived and designed the study. IK and KDH acquired funding. All authors reviewed and approved the final manuscript and agreed to be accountable for all aspects of the work.

## Supplementary Material

Supplemental data

Supporting data values

## Version 1 05/23/2024

In-Press Preview

## Version 2 06/24/2024

Electronic publication
