# Supplementary material for: Endothelial to mesenchymal Notch signaling regulates skeletal repair
Source: JCI Insight. 2024 May 23;9(12):e181073. doi: 10.1172/jci.insight.181073 (PMC11383173; doi:10.1172/jci.insight.181073)
Supplement: Supplemental data [file jciinsight-9-181073-s143.pdf]

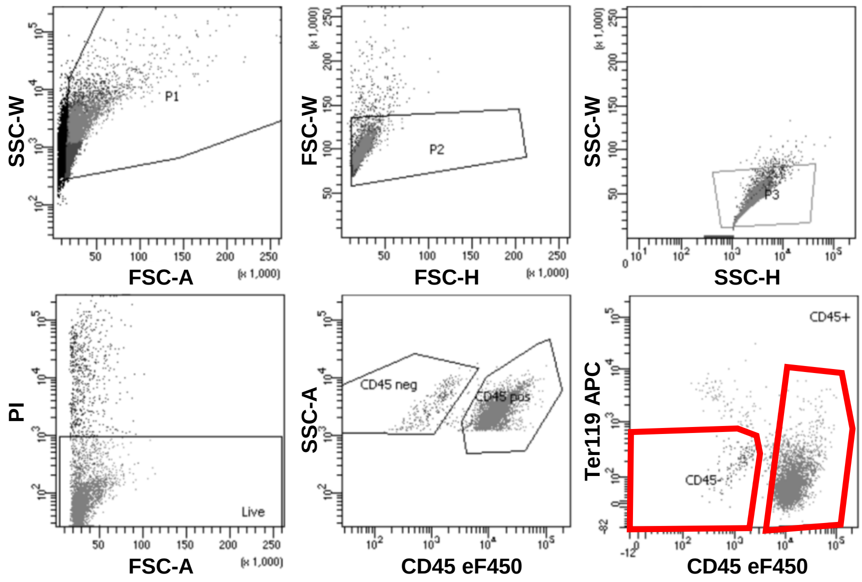

Supplemental Figure 1. Gating strategy for sorting to collect cells for scRNA-seq. Samples were stained for CD45 and Terr119 and sorted using a BD FACSSymphonyTM S6 Cell Sorter. Live, single cells negative for $CD45^{-}$ were collected to identify non-hematopoietic cells within periosteal samples, or $CD45^{+}$ cells were collected as hematopoietic cells.

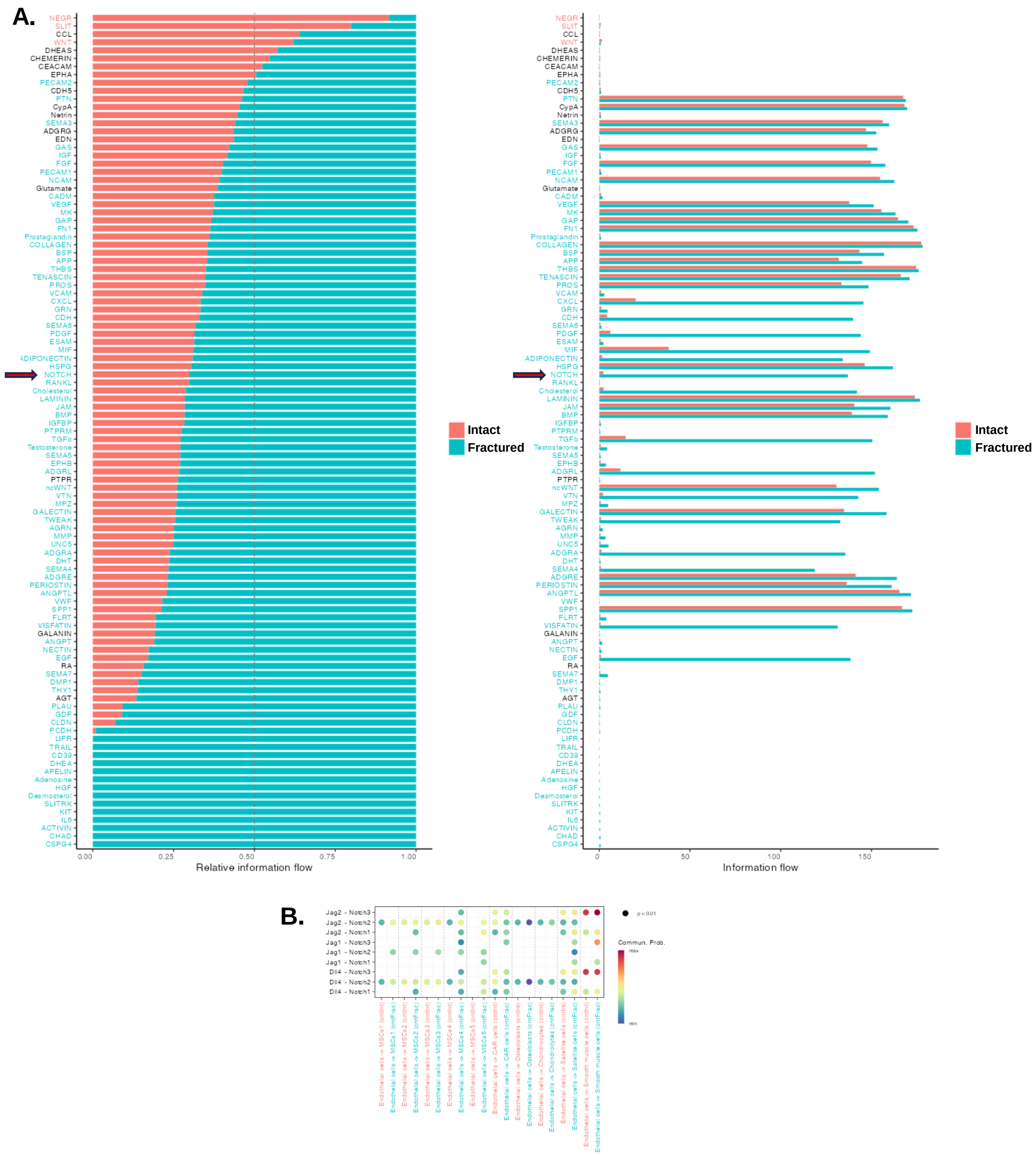

Supplemental Figure 2. Cell - cell interactions evaluated by CellChat (version 2.1.2). (A) Analysis of conserved and context-specific signaling pathways in Cre- intact sample compared to fractured Cre-. (B) Notch interactions of endothelial cells with mesenchymal clusters of Cre- intact and Cre- fractured sample.

A.

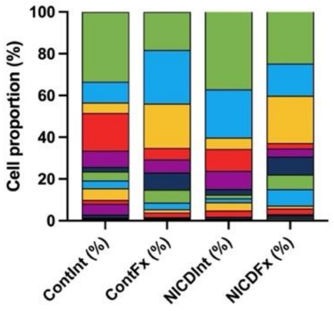

B.

Cluster	ContInt (%)	ContFx (%)	NICDInt (%)	NICDFx (%)
0	33.38	18.25	37.08	24.69
1	9.99	25.6	23.1	15.45
2	5	21.27	5.46	22.65
3	18.09	5.5	10.52	2.56
4	7.75	6.27	8.66	3.98
5	2.16	8.13	2.63	8.53
6	4.33	6.29	1.87	7
7	3.79	3.27	1.84	7.92
8	5.57	1.46	4	1.38
9	1.8	2.29	2.94	2.74
10	5.19	0.31	0.04	0.53
11	1.44	0.42	0.83	1.28
12	0.4	0.33	0.34	0.78
13	0.52	0.19	0.31	0.16
14	0.36	0.29	0.25	0.17
15	0.23	0.13	0.14	0.19

0 Endothelial cells
1 MSCs1
2 MSCs2
3 Cluster 3
4 MSCs3
5 Satellite cells
6 MSCs4
7 MSCs5
8 Chondrocytes
9 Smooth muscle cells
10 Cluster 10
11 CAR cells
12 Osteoblasts
13 Cluster 13
14 Cluster 14
15 Cluster 15

Supplemental Figure 3. (A) Proportion of cells within the Cre- and Cre+ intact and fractured samples of each cluster with numerical representation of cell frequency (**B**)

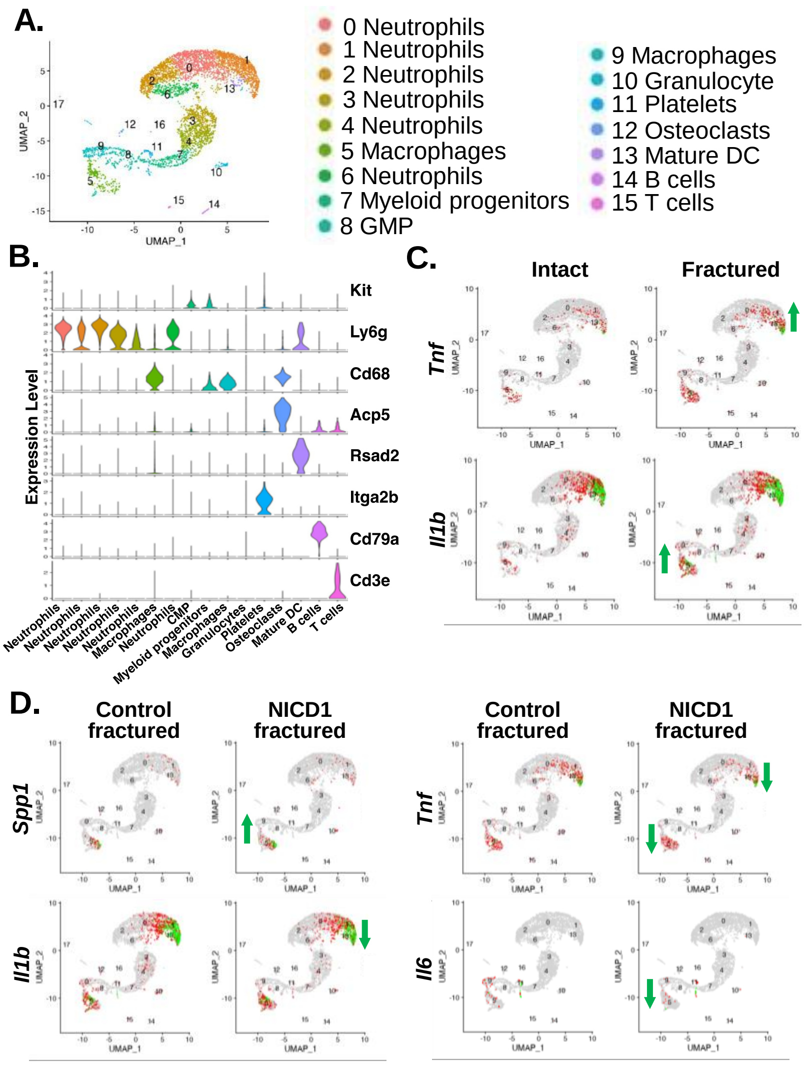

Supplemental Figure 4. Notch1 activation in osteoprogenitor cells decreases the proinflammatory transcriptional profile of hematopoietic cells. (A) Sorted $CD45^+$ cells were analyzed by 10X Genomics to determine transcriptional changes in the periosteum of intact and fractured control and in samples with NICD1 overexpression. Clusters were determined by unsupervised clustering using the Seurat package (version 4). **(B)** Violin plots of conserved genes within the clusters of hematopoietic cell populations. **(C)** We determined increased expression of *Tnf* and *Il1b* in neutrophils and macrophages upon femur fracture. **(D)** Cre^+ αSMACreER/NICD1 animals exhibited a significant decrease in the proinflammatory cytokines *Il6*, *Il1b*, and *Tnf* compared to Cre^- littermate controls at 3 dpf.

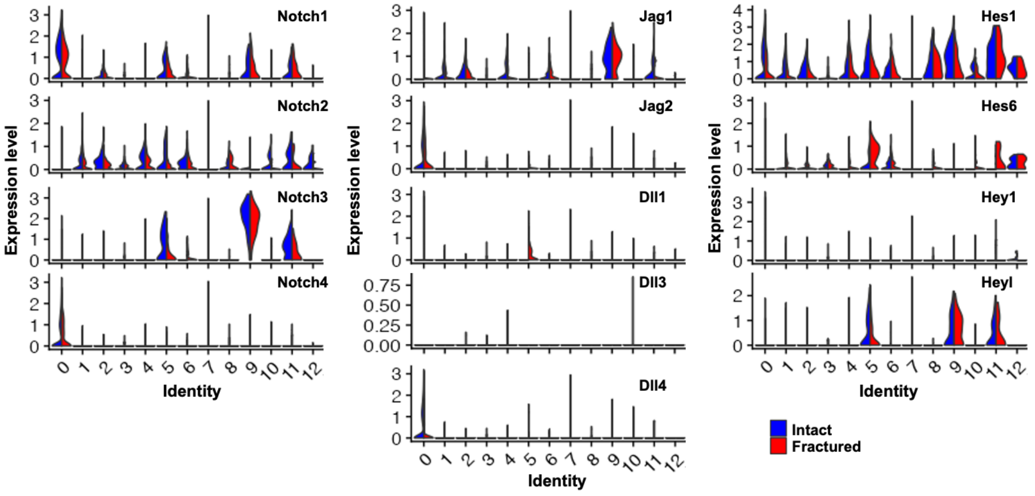

0 Endothelial cells
1 MSC 1
2 MSCs 2
4 MSCs 3
5 Satellite cells
6 MSCs 4
7 MSCs 5
8 Chondrocytes
9 Smooth muscle cells
10 Cluster 10
11 CAR cells
12 Osteoblasts

Supplemental Figure 5. Notch signaling genes in the periosteum. Violin plots presenting expression of Notch receptors, ligands, and the most highly expressed downstream signaling genes of integrated samples from intact and fractured Cre- groups.

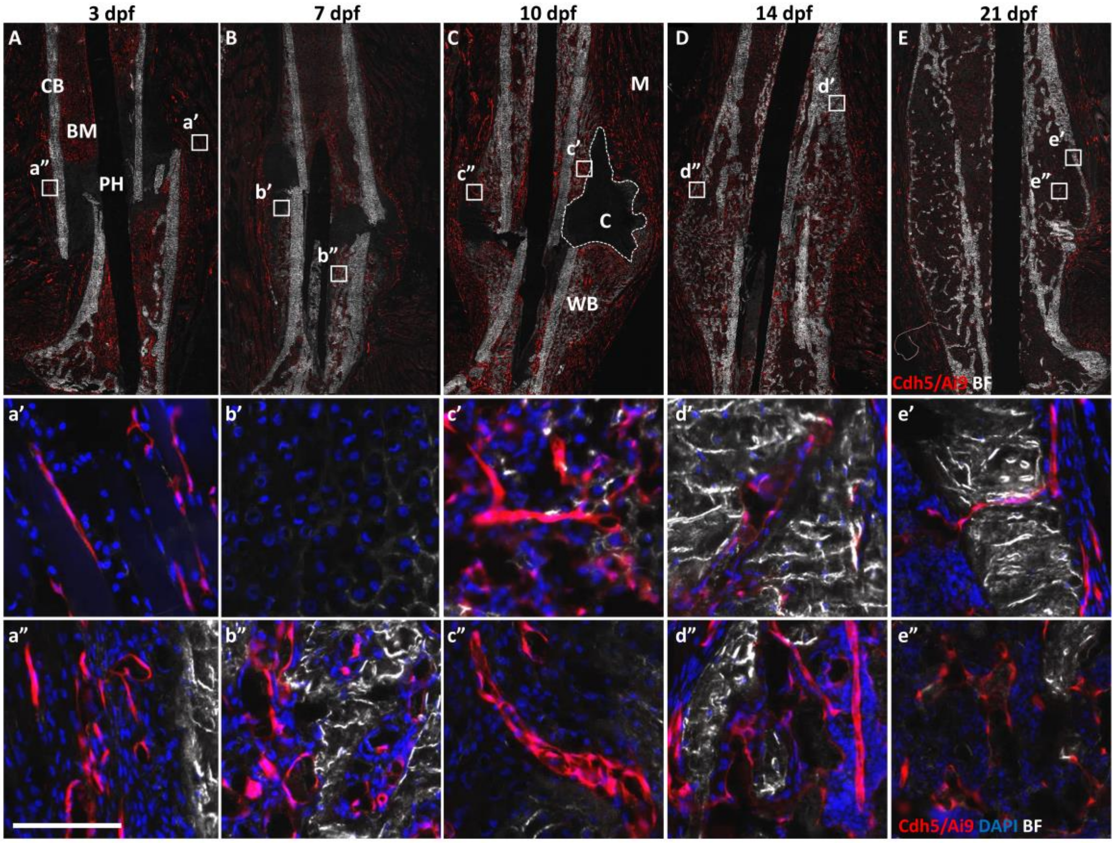

Supplemental Figure 6. Lineage tracing of Cdh5 ECs within the callus tissue. Cdh5CreER/Ai9 mice were injected with tamoxifen on the day of fracture to induce tdTomato expression and evaluate labeled ECs during fracture healing at 3, 7, 10, 14, and 21 dpf. Images present sections of the fractured femurs, with boxes indicating magnified images of callus and muscle tissue with labeled Cdh5/Ai9 ECs. Dashed line is presenting cartilage area with lack of Cdh5ER/Ai9 cells. CB-cortical bone, BM-bone marrow, PH-pin hole, WB-woven bone. Scale = 100 μm

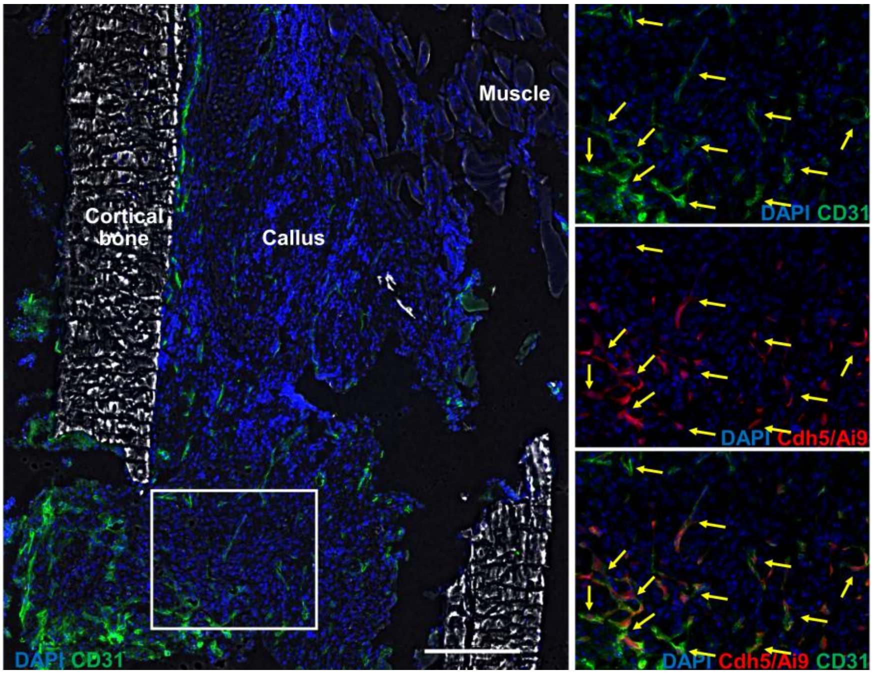

Supplemental Figure 7. Cdh5/Ai9 targets CD31^{+} cells. Cdh5CreER/Ai9 mice were injected with tamoxifen to induce tdTomato expression, and sections of fractured bone were stained for CD31 endothelial marker. tdTomato was co-expressed with CD31 marker, both staining ECs. Scale = 200 μm.

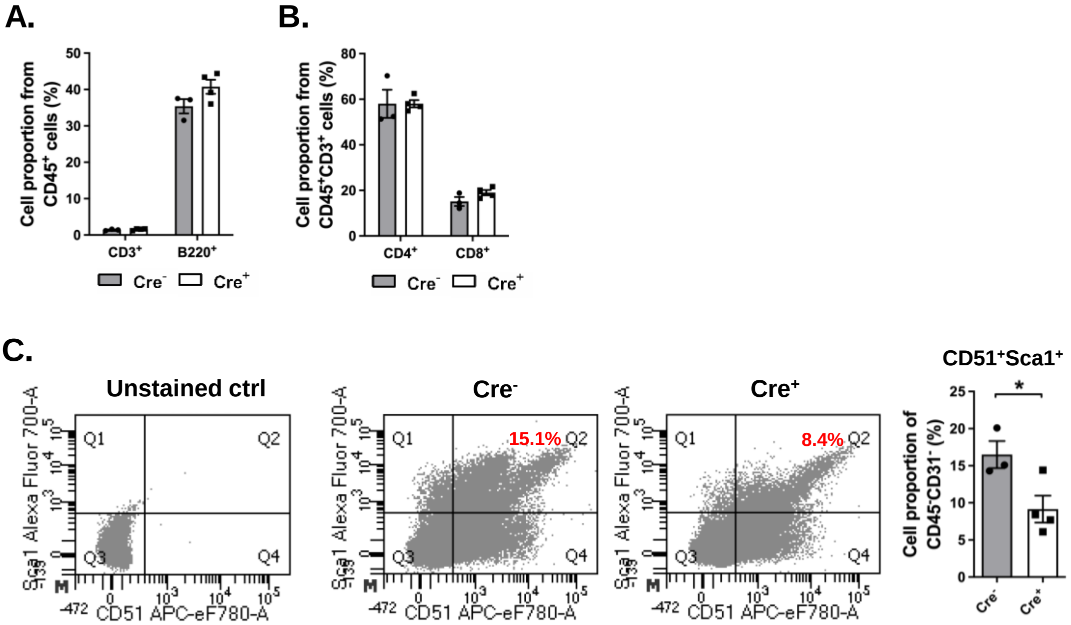

Supplemental Figure 8. Deletion of *Dll4* affects early callus healing. (**A**) To induce *Dll4* deletion, tamoxifen was injected into Cdh5CreER/Dll4Δ animals on the day of fracture and at 2 and 4 dpf. Cdh5Cre^{+}ER/Dll4Δ animals did not show changes in T- or B-cell proportions or (**B**) CD4^{+} and CD8^{+} cells within the bone marrow 5 days post first tamoxifen injection compared to Cre^{-} littermate controls. (**C**) At 5 dpf, *Dll4* deletion in ECs decreased the proportion of Sca1^{+}CD51^{+} periosteal cells. Cre^{-} n = 3, Cre^{+} n = 4, Unpaired t test. *$P < 0.05$.

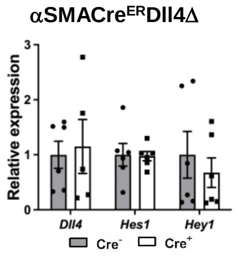

Supplemental Figure 9. Deletion of *Dll4* in osteoprogenitors has no effect on total callus *Dll4* expression. Deletion of *Dll4* in αSMACreER/Dll4Δ mice was induced by injecting tamoxifen into Cre^- and Cre^+ mice at 0, 2, and 4 dpf and evaluating the callus tissue on day 7 for expression of *Dll4* and the Notch downstream signaling genes *Hes1* and *Hey1*. n = 6, Unpaired t test.

Supplemental Table 1. List of pathways with significant cell-cell interactions

	Control intact	Control fractured	NICD1 intact	NICD1 fractured
1		ACTIVIN	ACTIVIN	ACTIVIN
2		Adenosine		Adenosine
3	ADGRA	ADGRA	ADGRA	ADGRA
4	ADGRE	ADGRE	ADGRE	ADGRE
5	ADGRG	ADGRG	ADGRG	ADGRG
6	ADGRL	ADGRL	ADGRL	ADGRL
7	ADIPONECTIN	ADIPONECTIN	ADIPONECTIN	ADIPONECTIN
8	AGRN	AGRN	AGRN	AGRN
9	AGT	AGT	AGT	AGT
10	ANGPT	ANGPT	ANGPT	ANGPT
11	ANGPTL	ANGPTL	ANGPTL	ANGPTL
12		APELIN		
13	APP	APP	APP	APP
14	BMP	BMP	BMP	BMP
15	BSP	BSP	BSP	BSP
16	CADM	CADM	CADM	CADM
17	CCL	CCL	CCL	CCL
18		CD39		CD39
19	CDH	CDH	CDH	CDH
20				
21	CDH5	CDH5	CDH5	CDH5
22	CEACAM	CEACAM	CEACAM	CEACAM
23		CHAD	CHAD	CHAD
24	CHEMERIN	CHEMERIN	CHEMERIN	CHEMERIN
25	Cholesterol	Cholesterol	Cholesterol	Cholesterol
26	CLDN	CLDN	CLDN	
27	COLLAGEN	COLLAGEN	COLLAGEN	COLLAGEN
28		CSPG4		CSPG4
29	CXCL	CXCL	CXCL	CXCL
30	CypA	CypA	CypA	CypA
31		Desmosterol	CysLTs	
32		DHEA		
33	DHEAS	DHEAS	DHEAS	DHEAS
34	DHT	DHT	DHT	DHT
35	DMP1	DMP1	DMP1	DMP1
36	EDN	EDN	EDN	EDN
37	EGF	EGF	EGF	EGF
38	EPHA	EPHA	EPHA	EPHA
39	EPHB	EPHB	EPHB	EPHB
40	ESAM	ESAM	ESAM	ESAM
41	FGF	FGF	FGF	FGF
42	FLRT	FLRT	FLRT	FLRT
43	FN1	FN1	FN1	FN1
44	GALANIN	GALANIN	GALANIN	GALANIN
45	GALECTIN	GALECTIN	GALECTIN	GALECTIN
46	GAP	GAP	GAP	GAP
47	GAS	GAS	GAS	GAS
48	GDF	GDF	GDF	GDF
49	Glutamate	Glutamate	Glutamate	Glutamate
50	GRN	GRN	GRN	GRN
51		HGF	HGF	HGF
52	HSPG	HSPG	HSPG	HSPG
53	IGF	IGF	IGF	IGF
54	IGFBP	IGFBP	IGFBP	IGFBP
55		IL6		
56	JAM	JAM	JAM	JAM
57		KIT		KIT
58	LAMININ	LAMININ	LAMININ	LAMININ
59		LIFR	LIFR	
60	MIF	MIF	MIF	MIF
61	MK	MK	MK	MK
62	MMP	MMP	MMP	MMP
63	MPZ	MPZ	MPZ	MPZ
64	NCAM	NCAM	NCAM	NCAM
65	ncWNT	ncWNT	ncWNT	ncWNT
66	NECTIN	NECTIN	NECTIN	NECTIN
67	NEGR	NEGR	NEGR	
68	Netrin	Netrin	Netrin	Netrin
69	NOTCH	NOTCH	NOTCH	NOTCH
70			NT	
71	PCDH	PCDH	PCDH	PCDH
72	PDGF	PDGF	PDGF	PDGF
73	PECAM1	PECAM1	PECAM1	PECAM1
74	PECAM2	PECAM2	PECAM2	PECAM2
75	PERIOSTIN	PERIOSTIN	PERIOSTIN	PERIOSTIN
76	PLAU	PLAU	PLAU	PLAU
77	PROS	PROS	PROS	PROS
78	Prostaglandin	Prostaglandin	Prostaglandin	Prostaglandin
79	PTN	PTN	PTN	PTN
80	PTPR	PTPR		PTPR
81	PTPRM	PTPRM	PTPRM	PTPRM
82	RA	RA	RA	RA
83	RANKL	RANKL	RANKL	RANKL
84	SEMA3	SEMA3	SEMA3	SEMA3
85	SEMA4	SEMA4	SEMA4	SEMA4
86	SEMA5	SEMA5	SEMA5	SEMA5
87	SEMA6	SEMA6	SEMA6	SEMA6
88	SEMA7	SEMA7	SEMA7	SEMA7
89	SLIT	SLIT	SLIT	SLIT
90		SLITRK		SLITRK
91	SPP1	SPP1	SPP1	SPP1
92	TENASCIN	TENASCIN	TENASCIN	TENASCIN
93	Testosterone	Testosterone	Testosterone	Testosterone
94	TGFb	TGFb	TGFb	TGFb
95	THBS	THBS	THBS	THBS
96	THY1	THY1	THY1	THY1
97		TRAIL		
98	TWEAK	TWEAK	TWEAK	TWEAK
99	UNC5	UNC5	UNC5	UNC5
100	VCAM	VCAM	VCAM	VCAM
101	VEGF	VEGF	VEGF	VEGF
102	VISFATIN	VISFATIN	VISFATIN	VISFATIN
103	VTN	VTN	VTN	VTN
104	VWF	VWF	VWF	VWF
105	WNT	WNT	WNT	WNT
n = pathways	89	103	94	95
n = shared pathways	86	86	86	86
Proportion of shared pathways	**96.63**	**83.50**	**91.49**	**90.53**

Supplemental Table 2. List of used antibodies and streptavidin

Antibody	Clone	Catalog number	Company	Dilution
CD45 eFluor 450	30-F11	48–0451	eBioscience	1:400
Ter119 APC	Ter-119	17–5921	eBioscience	1:400
Ter119 eFluor 450	TER-119	48–5921	eBioscience	1:402
CD51 biotin	RMV-7	13–0512	eBioscience	1:100
Sca1 Alexa fluor 700	D7	56–5981	eBioscience	1:100
CD90.2 BV605	53-2.1	740334	BD Bioscience	1:100
Dll4 APC	HMD4-1	130813	BioLegend	1:100
CD31 FITC	PECAM-1	11-0311	eBioscience	1:200
CD140a PE Cy7	APA5	25–1401	eBioscience	1:100
CD3 BV605	145-2C11	100351	eBioscience	1:200
CD4 PE	GK1.5	12-0041	eBioscience	1:200
CD8 biotin	53-6.7	13-0081	eBioscience	1:400
CD45 APC	30-F11	17–0451	eBioscience	1:400
B220 BV711	103255	103255	BioLegend	1:200
Streptavidin APC eFluor 780		47–4317	eBioscience	1:400
